# Probabilistic methods surpass parsimony when assessing clade support in phylogenetic analyses of discrete morphological data

**DOI:** 10.1111/pala.12330

**Published:** 2017-10-31

**Authors:** Joseph E. O'Reilly, Mark N. Puttick, Davide Pisani, Philip C. J. Donoghue

**Affiliations:** ^1^ School of Earth Sciences University of Bristol Life Sciences Building Tyndall Avenue Bristol BS8 1TQ UK; ^2^ Department of Earth Sciences The Natural History Museum Cromwell Road London SW7 5BD UK; ^3^ School of Biological Sciences University of Bristol Life Sciences Building Tyndall Avenue Bristol BS8 1TQ UK

**Keywords:** phylogenetic analysis, morphology, parsimony, Maximum Likelihood, Bayesian, Mk model

## Abstract

Fossil taxa are critical to inferences of historical diversity and the origins of modern biodiversity, but realizing their evolutionary significance is contingent on restoring fossil species to their correct position within the tree of life. For most fossil species, morphology is the only source of data for phylogenetic inference; this has traditionally been analysed using parsimony, the predominance of which is currently challenged by the development of probabilistic models that achieve greater phylogenetic accuracy. Here, based on simulated and empirical datasets, we explore the relative efficacy of competing phylogenetic methods in terms of clade support. We characterize clade support using bootstrapping for parsimony and Maximum Likelihood, and intrinsic Bayesian posterior probabilities, collapsing branches that exhibit less than 50% support. Ignoring node support, Bayesian inference is the most accurate method in estimating the tree used to simulate the data. After assessing clade support, Bayesian and Maximum Likelihood exhibit comparable levels of accuracy, and parsimony remains the least accurate method. However, Maximum Likelihood is less precise than Bayesian phylogeny estimation, and Bayesian inference recaptures more correct nodes with higher support compared to all other methods, including Maximum Likelihood. We assess the effects of these findings on empirical phylogenies. Our results indicate probabilistic methods should be favoured over parsimony.

The goal of reconstructing an holistic Tree of Life has been envisaged since the inception of evolutionary theory. This entails not only the use of molecular phylogenetics to determine the inter‐specific relationships between extant taxa, but also the restoration of extinct branches to the Tree of Life. For the majority of extinct species, this can only be achieved through phylogenetic analysis of morphological data. Parsimony has dominated the phylogenetic analysis of morphological data but its hegemony is now challenged by model‐based phylogenetic methods that attempt to approach the realism of models of evolution developed for molecular evolution (Lewis 2001).

Simulation‐based studies have shown that parsimony is less accurate than Bayesian analysis for phylogenetic inference with morphological data (Wright & Hillis 2014; O'Reilly *et al*. 2016; Puttick *et al*. 2017). Previous studies (O'Reilly *et al*. 2016; Puttick *et al*. 2017) treated the 50% majority rule consensus tree constructed from a sample of trees from the posterior distribution (hereafter the Bayesian tree) as optimality trees (following Holder *et al*. 2008) and compared them directly to the optimal Maximum Likelihood and Maximum Parsimony trees. However, because Bayesian trees have intrinsic support values (posterior probabilities) some have argued that they should be compared to bootstrapped Maximum Likelihood trees (Huelsenbeck *et al*. 2001), even though posterior probabilities and bootstrap proportions are neither interchangeable nor directly comparable (Douady *et al*. 2003). Brown *et al*. (2017) showed that, when clade support is considered, Maximum Likelihood and Bayesian implementations of the Mk model are effectively indistinguishable in terms of topological accuracy.

Here we develop upon previous studies to incorporate measures of clade support in the investigation of the performance of parsimony relative to Maximum Likelihood and Bayesian implementations of the Mk model in the phylogenetic analyses of morphological data. An initial comparison of the accuracy and resolution of different methods is achieved through the use of simulated data. We take a simulation‐based, rather than an empirical approach since the performance of phylogenetic methods in tree estimation can only be assessed when the tree is known; this is never the case for empirical data. Empirical analyses are performed to demonstrate the influence of methods on the testing of phylogenetic hypotheses. We compare clade support using standard methods, with posterior probabilities obtained from posterior samples of Bayesian trees and non‐parametric bootstrap proportions calculated for most‐parsimonious and Maximum Likelihood trees, collapsing poorly‐supported (nodes with < 0.5 posterior probability, or present in < 50% of bootstrap replicates) nodes in trees constructed with each method. We find that parsimony has low accuracy compared to both Bayesian and Maximum Likelihood implementations of the Mk model. However, the Bayesian implementation still achieves higher support values on correct nodes than does the Maximum Likelihood implementation. Based on these results, we conclude that a Bayesian implementation of the Mk model should be preferred over the Maximum Likelihood implementation when analysing categorical morphological data. Nevertheless, both implementations are superior to parsimony when analysing categorical morphological data.

## Material and method

### Simulated data

For all analyses, we estimated topologies and support values using datasets of 100, 350, and 1000 characters from Puttick *et al*. (2017), simulated on both the asymmetrical and symmetrical phylogenies; these variables allowed us to explore the impact of both character matrix size and tree symmetry on phylogeny estimation. We generated 1000 independent datasets for each tree and character set size. These data were simulated to ensure that they matched an empirical distribution of homoplasy. To reduce potential biases in favour of the use of the Mk model for phylogenetic inference (regarding Parsimony, see O'Reilly *et al*. 2016) we simulated our data using a model with more parameters than the Mk model: the HKY + Γ_continuous_ model of molecular evolution (Hasegawa *et al*. 1985), with the transition to transversion ratio parameter *k* fixed to a value of 2. This approach to simulation enforces the violation of the assumption of the Mk model that a transition between any two character states is equally probable. The substitution rate of each dataset and the shape of the gamma distribution of character‐wise rate heterogeneity were sampled independently from an exponential distribution of mean 1, ensuring a reasonable level of rate heterogeneity between replicate datasets and between the constituent characters of each replicate matrix. The choice of rate parameters in our simulation framework is validated by our ability to easily simulate matrices with empirical levels of homoplasy. To ensure data did not collapse into the Mk model and therefore unintentionally provide a benefit when applying the Mk model for phylogenetic inference, we applied an unequal stationary distribution of **π** = [0.2, 0.2, 0.3, 0.3] in the HKY model. Fixing the stationary distribution in this manner enforces violation of the assumptions of the Mk model that the limiting distribution of character states is equal and that transitions between any two character states are equally probable. A mixture of binary and multistate characters was simulated, with binary characters obtained through the R/Y (purine/pyrimidine) recoding of molecular data as character states of 0 or 1, resulting in the elimination of the transition–transversion ratio for these characters and an effective stationary distribution of **π** = [0.4, 0.6], which is a violation of the assumption of the Mk model that character states exhibit an even limiting distribution. Multistate characters were obtained by recoding nucleotide data as character states of 0, 1, 2, or 3, depending on the nucleotide present at each terminal. The final ratio of binary to multistate characters in each matrix was 55:45, based on the mean ratio observed in empirical data (Guillerme & Cooper 2016). For each of the 100, 350, and 1000 character sets we generated 1000 matrices that, in total, exhibited a distribution of homoplasy approximating that reported by Sanderson & Donoghue (1996).

Goloboff *et al*. (2017) have criticized this approach to simulating morphology‐like datasets on the basis that our generating trees encompass only contemporaneous taxa, assume that evolutionary rates are constant across time and the tree, and that our measure of biological realism, the spread of homoplasy exhibited by datasets, is inadequate. However, our experiments do not attempt to simulate non‐contemporary taxa or address the problem of missing data, qualities of palaeontological data that are of a level of complexity that is beyond the current debate. Goloboff *et al*. (2017, fig. 1A) demonstrated that our simulated data broadly achieve their preferred measure of biological realism. Our review of their datasets indicates that, while Goloboff *et al*. (2017) drew characters from an empirically realistic global distribution of homoplasy, their simulated datasets are not individually empirically realistic, with many matrices dominated by characters with very high consistency and an unrealistically small proportion of characters exhibiting high levels of homoplasy. The datasets simulated by Goloboff *et al*. (2017) have qualities that strongly bias in favour of parsimony phylogenetic inference, and implied‐weights parsimony in particular, as the presence of large numbers of characters that are congruent with the tree allows implied weights to increase the power of these ‘true’ congruent characters. This effect will not be possible when increased levels of homoplasy are present or when the true tree is unknown (as is the case for all empirical datasets).

For each of our datasets, we estimated trees using the Maximum Likelihood and Bayesian implementations of the Mk model, and equal‐weights parsimony, in RAxML (Stamatakis 2014), MrBayes (Ronquist *et al*. 2012) and TNT (Goloboff *et al*. 2008), respectively. Bayesian phylogenetic methods that use MCMC sampling result in a distribution of trees by design, Maximum Likelihood invariably recovers a single tree that maximizes the likelihood function, and parsimony recovers one or several equally most‐parsimonious trees. In the event of the recovery of multiple equally most‐parsimonious trees, a majority rule consensus tree was constructed from this set. Majority rule consensus trees were constructed from the posterior sample of trees obtained with the Bayesian implementation of the Mk model. Alternative consensus tree methods are available, each with their attendant advantages and disadvantages (Heled & Bouckaert 2013; Holder *et al*. 2008); we selected the majority rule consensus method to maintain comparability among inference frameworks as other consensus tree construction methods are not universally applicable. The Robinson–Foulds (1981) distance relative to the generating tree was calculated for each of the trees estimated across all three inference frameworks (parsimony, Maximum Likelihood and Bayesian analysis). The Robinson–Foulds distance is well understood and rooted in set theory, as it represents the symmetric difference between the sets of all the clades in each considered tree. The Robinson–Foulds distance between two trees is calculated as the sum of the clades found in the first tree but not in the second plus the sum of clades found in the second tree but not the first one. Accordingly, smaller Robinson–Foulds distances are characteristic of more accurate phylogenetic trees (i.e. trees that do not disagree with the generating tree). A drawback of the Robinson–Foulds metric is that small distances are expected for unresolved trees; that is, they can be achieved without precision, by consensus trees lacking resolution. Accordingly, to qualify whether accuracy is achieved with or without precision, we also measure resolution (i.e. number of nodes in the recovered tree) and consider it in our interpretations.

For each method, we integrated the effect of support on phylogenetic accuracy collapsing nodes that had < 50% support and re‐calculating the Robinson–Foulds distances relative to the generating tree (Robinson & Foulds 1981). In these analyses, ≥ 50% support is defined by a node with a posterior probability of ≥ 0.5, or a node that is present in ≥ 50% of bootstrap replicates. The trees estimated in the Bayesian framework already represent the 50% majority rule consensus of the posterior distribution of trees, and so these trees are identical before and after assessing support. For the Maximum Likelihood and parsimony analyses, we estimated clade support using non‐parametric bootstrapping (Felsenstein 1985). We obtained 250 bootstrap replicates for each simulated dataset in both parsimony and Maximum Likelihood frameworks, using TNT (Goloboff *et al*. 2008) and RAxML (Stamatakis 2014), respectively. We collapsed branches on the parsimony and Maximum Likelihood trees with < 50% bootstrap support. Support for clades in trees estimated from Bayesian analysis was assessed using posterior probability.

### Empirical data

Puttick *et al*. (2017) re‐analysed four published morphological matrices (Hilton & Bateman 2006; Sutton *et al*. 2012; Nesbitt *et al*. 2013; Luo *et al*. 2015) using Bayesian, Maximum Likelihood and parsimony frameworks to identify the influence that each method has on the support for published hypotheses. We analysed these datasets again using the same three phylogenetic inference frameworks in addition to estimating non‐parametric bootstrap support for clades in these four estimated topologies with both parsimony and the Maximum Likelihood implementation of the Mk model (support being obtained intrinsically within this Bayesian framework) and collapsed nodes with less than 50% support on the Maximum Likelihood and parsimony trees. As with the simulated datasets, non‐parametric bootstrapping was performed in TNT and RAxML for parsimony and Maximum Likelihood, respectively, with 250 replicates obtained. Our aim is to determine whether the phylogenetic conclusions drawn in the original studies were contingent on the phylogenetic method employed.

## Results

### Simulations

In all of our analyses, competing phylogenetic methods exhibited greater accuracy when reconstructing data from the symmetrical tree compared to data from the asymmetrical tree. For the datasets derived from the asymmetrical generating tree, the Bayesian and Maximum Likelihood implementations of the Mk model outperformed parsimony in terms of accuracy (Table 1).

**Table 1:** The median and 95% quantile of Robinson–Foulds distances for the asymmetrical and symmetrical phylogenies with 100, 350 and 1000 characters

	Bayesian asymmetrical	ML asymmetrical	Parsimony asymmetrical	Bayesian symmetrical	ML symmetrical	Parsimony symmetrical
100 majority rule	29 (23–35)	47 (31–59)	35 (27–48.02)	7 (1–25)	7 (1–43.05)	7 (1–26)
100 ≥ 50% branch support	29 (23–35)	30 (24–34)	30 (26–32)	7 (1–25)	7 (2–24.02)	9 (3–27)
350 majority rule	20 (12–30)	25 (13–55)	28 (18–37)	1 (1–15.02)	1 (1–23)	1 (1–15.02)
350 ≥ 50% branch support	20 (12–30)	20 (14–31)	24 (19–30)	1 (1–15.02)	1 (1–15)	1 (1–17)
1000 majority rule	9 (3–26)	11 (3–43)	18 (9–30)	1 (1–3.02)	1 (1–5)	1 (1–3.02)
1000 ≥ 50% branch support	9 (3–26)	10 (4–27)	17 (10–28)	1 (1–3.02)	1 (1–4)	1 (1–4)

ML, Maximum Likelihood

**Figure 1:**
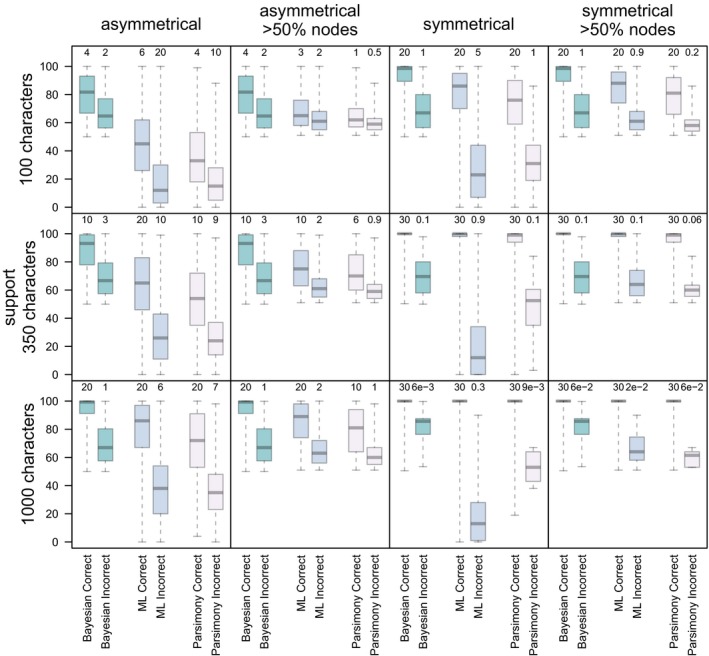
The distribution of support values on accurate and inaccurate nodes using all methods with simulated datasets summarizing support for all nodes, and nodes that have above 50% accuracy only. The boxplots show the support for accurate and inaccurate nodes across all trees, and the numbers above show the mean resolution (number of nodes) of the 1000 trees. ML, Maximum Likelihood. Colour online.

Support values for nodes were generally higher when nodes were accurately reconstructed, and this was more pronounced in analyses of datasets generated from the asymmetrical tree compared to those derived from the symmetrical generating tree (Fig. 1). Bayesian methods reconstructed the highest number of accurate nodes and had higher support on these nodes compared to inaccurate nodes, and accurate nodes recovered by alternative methods.

### Effects of considering support values

**Figure 2:**
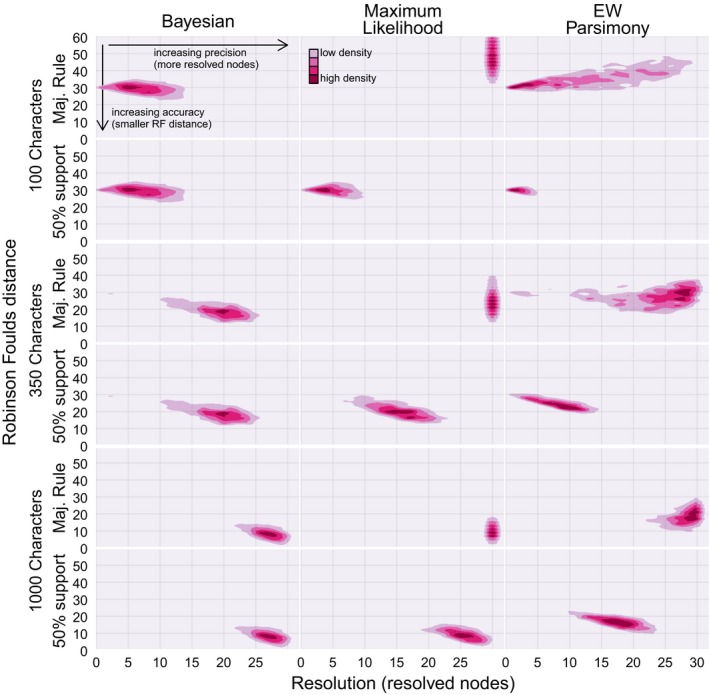
Contour plots of resolution against Robinson–Foulds distance for data (100, 350, 1000 characters) simulated on the asymmetrical tree before and after nodes with less than 50% support are collapsed. Performance of Maximum Likelihood and Parsimony improves after these nodes are collapsed, but both Maximum Likelihood and Bayesian surpass parsimony in accuracy. EW, equal weights. Colour online.

**Figure 3:**
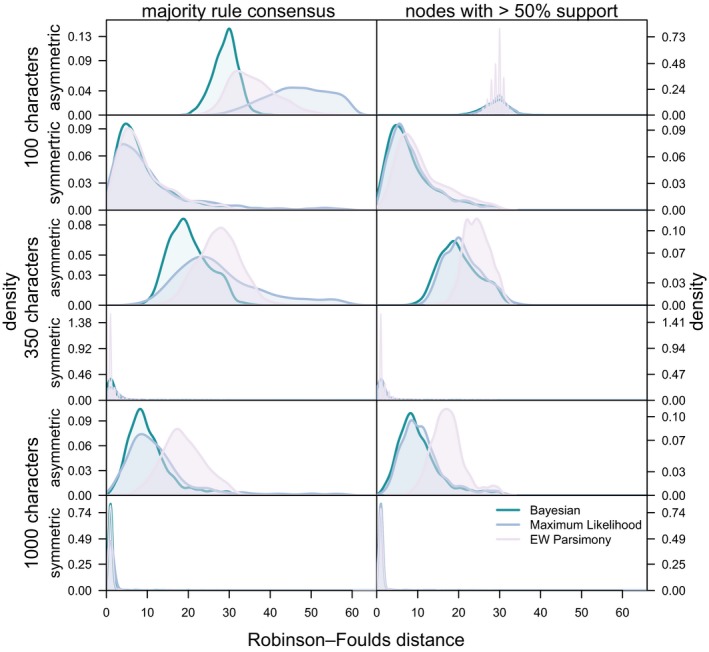
Distribution of Robinson–Foulds distance for all datasets on the asymmetrical and symmetrical phylogenies. After collapsing nodes with < 50% support Maximum Likelihood and Bayesian achieve similar values, and both methods out‐perform parsimony. EW, equal weights. Colour online.

**Figure 4:**
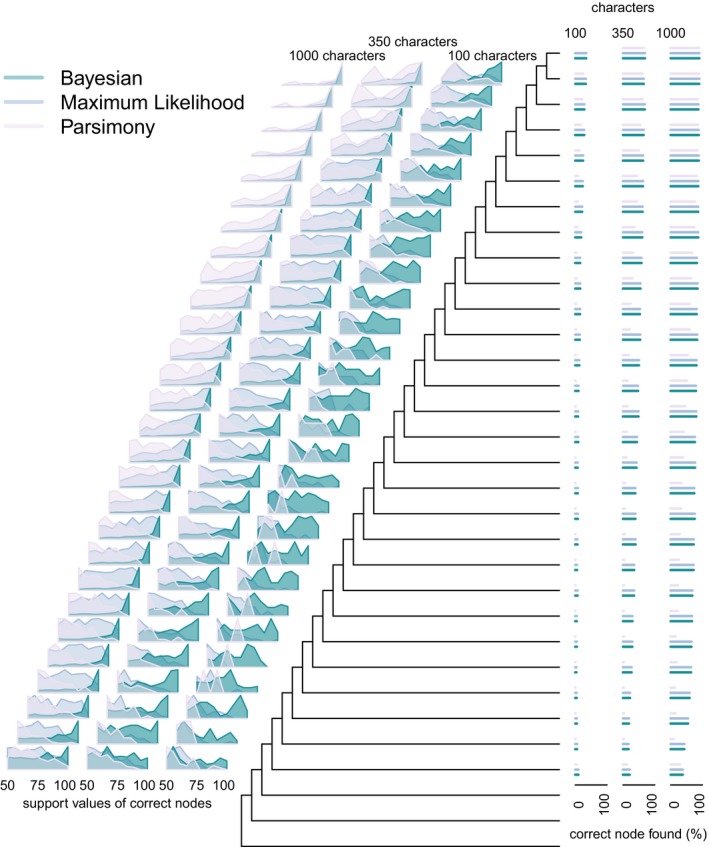
The degree of accuracy (barplots at tips) and support (density plots at nodes) for each method and all datasets on the asymmetrical phylogeny. Performance of all methods increases with dataset size, and Bayesian methods tend to find higher support for correct nodes with datasets containing 100 characters. Colour online.

Both the Maximum Likelihood implementation of the Mk model and parsimony produced trees with increased accuracy after nodes with < 50% support were collapsed. For the asymmetrical tree, the accuracy of the Maximum Likelihood and Bayesian implementations of the Mk model overlap, but parsimony is the least accurate method (Figs 2, 3, 4; Table 1). Similar results were obtained from analysis of the data derived from the symmetrical generating tree (Table 1). For both the symmetrical and asymmetrical trees, accuracy increases with dataset size, and the Bayesian and Maximum Likelihood Mk implementations achieve very high accuracy at datasets with 1000 characters.

Bayesian majority rule consensus trees were generally more resolved than the 50% support trees obtained from Maximum Likelihood topology estimates; the 50% support trees estimated using parsimony were the most conservative (Table 2). This trend was also observed in the results of analyses of 100 character datasets derived from the symmetrical generating tree. However, all methods yielded more fully resolved 50% support trees based on 350 and 1000 character datasets.

**Table 2:** The median and 95% quantile of resolution for the asymmetrical and symmetrical phylogenies with 100, 350, and 1000 characters

	Bayesian asymmetrical	ML asymmetrical	Parsimony asymmetrical	Bayesian symmetrical	ML symmetrical	Parsimony symmetrical
100 majority rule	8 (1–15)	30 (30–30)	16 (2–28)	27 (7–30)	30 (30–30)	27 (6.98–30)
100 ≥ 50% branch support	8 (1–15)	6 (1–12)	3 (1–7)	27 (7–30)	26 (7.98–30)	22 (4.98–28)
350 majority rule	19 (4–25)	30 (30–30)	24 (4–30)	30 (16–30)	30 (30–30)	30 (16–30)
350 ≥ 50% branch support	19 (4–25)	15 (4–23)	9 (2–15)	30 (16–30)	30 (16–30)	30 (14–30)
1000 majority rule	26.5 (8–30)	30 (30–30)	28 (7.98–30)	30 (28–30)	30 (30–30)	30 (28–30)
1000 ≥ 50% branch support	26.5 (8–30)	25 (8–29)	17 (4.98–24)	30 (28–30)	30 (28–30)	30 (27.98–30)

ML, Maximum Likelihood

**Figure 5:**
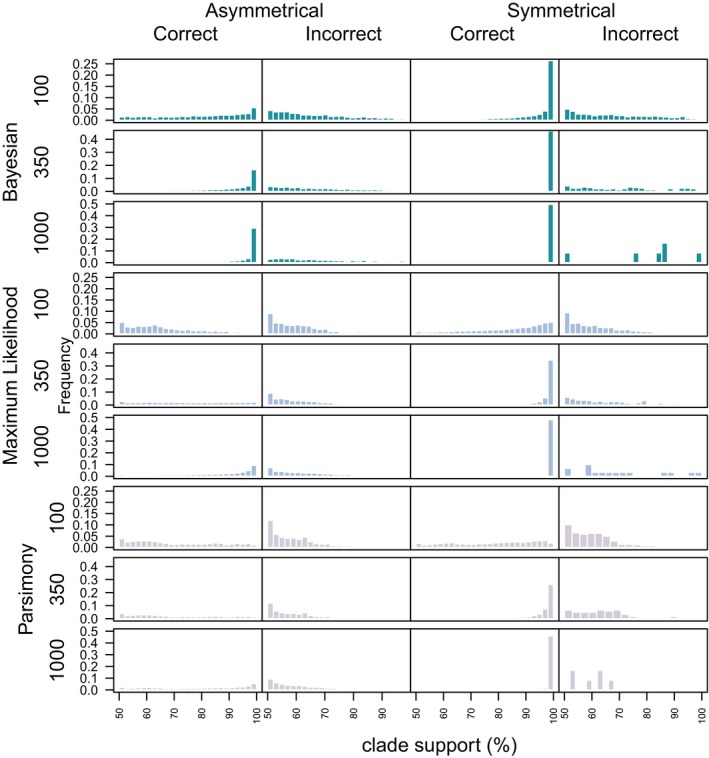
Frequency of clade support for accurate and inaccurate branches for all methods and dataset sizes. Bayesian and Maximum Likelihood methods show higher clade support for correct nodes, and lower support for inaccurate nodes with increasing dataset size. Bayesian trees have highest support compared to all other methods on the asymmetrical and symmetrical phylogenies. Colour online.

The Bayesian Mk implementation resolves a higher number of correct nodes across all analyses (Fig. 1), and these have higher support compared to the Maximum Likelihood implementation and parsimony (Fig. 5). However, overall, the Bayesian implementation resolves more inaccurate nodes than does either the Maximum Likelihood Mk implementation or parsimony. Median support for correct nodes is higher in Bayesian, as opposed Maximum Likelihood analyses, across all dataset sizes, and this trend is particularly evident on the asymmetric trees (Fig. 1). The median support for correct nodes is higher than for incorrect nodes in trees derived from the Bayesian Mk implementation, and over all methods (Fig. 1). Bayesian support is higher for incorrect nodes compared to Maximum Likelihood and parsimony (Fig. 1). There is a clear difference in the levels of support for correct and incorrect nodes in the Bayesian trees, with correct nodes generally achieving greater than 80% support and incorrect nodes exhibiting less than 80% support. Applying an 80% threshold for dataset sizes of 350 and above, the Bayesian Mk implementation resolves only correct nodes (Fig. 1); the Maximum Likelihood Mk implementation and parsimony do not match this level of support.

### Empirical data

**Figure 6:**
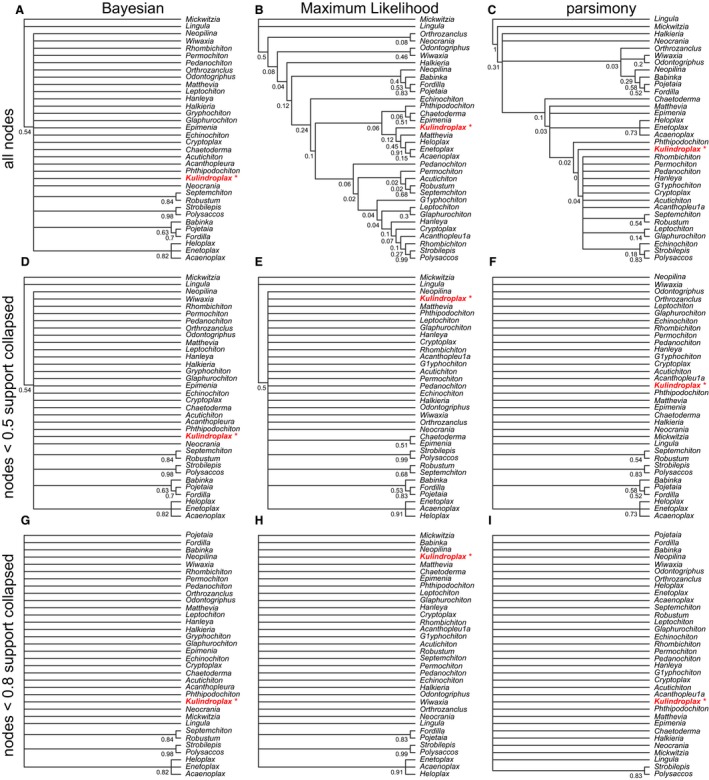
Bayesian, Maximum Likelihood and parsimony topologies of the data from Sutton *et al*. (2012). The optimal tree with node support is shown for each method (A–C) as well as the topologies that are produced after nodes with < 0.5 support are collapsed to polytomies (D–F), and when nodes with < 0.8 support are collapsed (G–I). After these nodes are collapsed, no method supports a crown‐group mollusc affiliation for *Kulindroplax*. Colour online.

**Figure 7:**
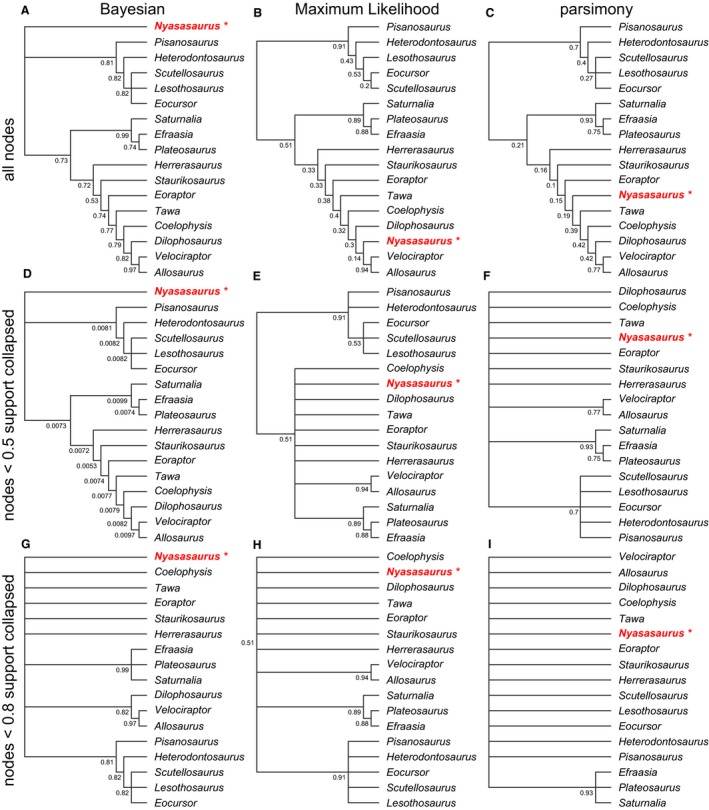
Bayesian, Maximum Likelihood, and parsimony topologies of the data from Nesbitt *et al*. (2013). The optimal tree with node support is shown for each method (A–C) as well as the topologies that are produced after nodes with < 0.5 support are collapsed to polytomies (D–F), and when nodes with < 0.8 support are collapsed (G–I). Only Bayesian (A) recovers the major clades of Dinosauria (Ornithischia, Saurischia, Sauropoda, Theropoda). Colour online.

There were substantial differences between the empirical topologies derived from the Maximum Likelihood Mk implementation and the parsimony framework, before and after the collapse of nodes with < 50% support (Figs 6, 7; O'Reilly *et al*. 2017, figs S1, S2). Smaller datasets showed the largest differences in topology and the placement of key taxa whether support is accounted for by collapsing poorly supported nodes or not.

For the smaller datasets, there was congruence between topologies estimated with different methods after poorly supported nodes were collapsed. *Kulindroplax*, from Sutton *et al*. (2012), was not supported as a crown‐mollusc with any method when < 50% supported nodes are collapsed (Fig. 6). This contrasts with the crown‐mollusc affinity of *Kulindroplax* in the Maximum Likelihood Mk estimated and most‐parsimonious trees (Fig. 6). A similar pattern was observed in the topologies estimated from the data of Hilton & Bateman (2006). Both the Maximum Likelihood implementation of the Mk model and the parsimony framework supported the anthophyte hypothesis in the respective optimal trees. After collapsing nodes with < 50% support, both methods yielded topologies that are more congruent with the Bayesian majority rule consensus tree, with a polytomy uniting, but not differentiating between, gymnosperms, seed ferns and angiosperms (O'Reilly *et al*. 2017, fig. S1).

Collapsing poorly supported nodes in trees estimated from the empirical datasets had less impact on the placement of key taxa in datasets with larger numbers of characters. Within the majority rule consensus tree obtained from the Bayesian implementation of the Mk model, *Nyasaurus* was resolved in a polytomy with the major clades of Dinosauria (Saurischia, Ornithischia; but see Baron *et al*. 2017), as was shown by Nesbitt *et al*. (2013). *Nyasasurus* was also resolved as a member of Dinosauria in the Maximum Likelihood Mk estimate and most‐parsimonious trees; this conclusion was not impacted by collapsing nodes with less than 50% bootstrap support (Fig. 7). However, there were changes in the certainty of placement of *Nyasasaurus* after support was assessed, collapsing from membership of Theropoda to Saurischia in the Maximum Likelihood Mk analyses, and from Theropoda to Dinosauria in parsimony analyses. Neither the Maximum Likelihood Mk implementation nor parsimony recovered Saurischia, Ornithischia, Theropoda or Sauropoda, though these clades were resolved by the Bayesian Mk implementation (Fig. 7). A similar pattern is seen in the re‐analysis of the dataset from Luo *et al*. (2015). All trees, before and after accounting for support in the final topology, resolved *Haramiyavia* outside crown‐Mammalia and the multiturberculates (O'Reilly *et al*. 2017, fig. S2).

Analyses of the smallest dataset (34 characters, 48 taxa), from Sutton *et al*. (2012), recovered only seven nodes in the Bayesian majority rule consensus tree, but more resolution was achieved in the Maximum Likelihood Mk estimate (32 nodes) and most‐parsimonious trees (17 nodes). After collapsing poorly‐supported nodes, a similar level of resolution was achieved by all methods: Maximum Likelihood Mk implementation (8 nodes) and parsimony (6 nodes). This contrasts with the results obtained from analyses of the other, larger empirical datasets where, after collapsing nodes with less than 50% support, the Bayesian Mk implementation consistently yielded trees with the greatest resolution: a pattern opposite to that seen in comparison of the optimal trees derived from the three methods of phylogenetic inference (Table 3).

**Table 3:** Resolution of the three methods on empirical trees before and after nodes with less than 50% support are collapsed

		Bayesian	Maximum Likelihood	Parsimony
Sutton (34 taxa, 48 characters)	Optimal tree	7	32	17
	Collapsed tree	7	8	6
Hilton (48 taxa, 82 characters)	Optimal tree	28	46	38
	Collapsed tree	28	15	13
Nesbitt (82 taxa, 413 characters)	Optimal tree	72	80	72
	Collapsed tree	72	63	47
Luo (114 taxa, 497 characters)	Optimal tree	92	112	109
	Collapsed tree	92	69	69

## Discussion

After incorporating estimates of node support, Parsimony is outperformed by both Maximum Likelihood and Bayesian implementations of the Mk model, providing further support for the use of stochastic models of character change in morphological data analyses (Wright & Hillis 2014; O'Reilly *et al*. 2016; Puttick *et al*. 2017). As shown by Brown *et al*. (2017), Bayesian and Maximum Likelihood implementations of the Mk model achieve similar levels of accuracy after collapsing weakly‐supported branches on Maximum Likelihood trees (Fig. 2). However, after node support is considered, the Bayesian Mk implementation recovers more correct nodes with higher support than does the Maximum Likelihood implementation, and support values on correct nodes are highest for the Bayesian implementation. Though bootstrapping increases the accuracy of the Maximum Likelihood Mk implementation to a level similar to the Bayesian implementation (Brown *et al*. 2017), Bayesian posterior probabilities are still higher for correct nodes.

### Incorporating support, probabilistic methods out‐perform parsimony, and the accuracy of ML improves

In line with previous results, probabilistic methods that implement the Mk model (Maximum Likelihood and Bayesian phylogenetics) achieve higher accuracy than does parsimony (Wright & Hillis 2014; O'Reilly *et al*. 2016; Puttick *et al*. 2017). Similar observations have been made in analyses of molecular data using probabilistic versus parsimony methods (Felsenstein 1978).

After considering support values, the Bayesian and Maximum Likelihood implementations of the Mk model achieve similar levels of accuracy (Brown *et al*. 2017) but the Bayesian implementation remains superior to Maximum Likelihood in a number of ways. The Bayesian implementation recovers more nodes with higher support compared to the Maximum Likelihood implementation, but the Bayesian method also recovers more incorrect nodes overall than does Maximum Likelihood (Fig. 5). Thus, the Maximum Likelihood implementation also produces a more topologically conservative tree, as it recovers fewer nodes overall (Fig. 1), which inevitably equates to improved Robinson–Foulds scores (Fig. 2). Support on the remaining correct nodes is not as high in trees derived using the Maximum Likelihood Mk implementation as it is for the Bayesian implementation; this trend is particularly pronounced at posterior probabilities of 0.8 and above in which Bayesian only recovers correct nodes.

As the number of analysed characters increases, the accuracy and resolution of all methods also increase, irrespective of tree symmetry. Despite this, the Maximum likelihood and Bayesian implementations of the Mk model still outperform Parsimony when a large number of characters are analysed. This general trend of improved accuracy and resolution should be expected from the stochastic models as they are statistically consistent, but the relatively accurate performance of Parsimony is less expected. These results suggest that no matter which method is applied to a dataset, it should be a goal for morphological datasets to include as many characters as possible if the most accurate estimates of topology are to be obtained.

Here, a stochastic Markov process was used to simulate categorical character data, facilitating a comparison of the efficacy of probabilistic (Bayesian, Maximum Likelihood) and non‐probabilistic models (parsimony) of evolution when morphological data are analysed. To ensure that our data generation process did not favour the probabilistic Mk model we enforced violation of the assumptions of this model in the simulation procedure, resulting in a suitable level of model misspecification when those data were analysed with the Mk model. However, it could be argued that the use of a stochastic Markov process to generate data will bias results toward the preference for the use of a stochastic Markov model for phylogenetic inference. Further, the stochastic process used for simulation assumes that the evolutionary rates of different characters are independent, in addition to assuming that the rates of evolution at different characters across the tree are proportional. However, the stochastic model we employ to generate data does produce realistic distributions of morphological characters at the tips of the tree, demonstrating that this approach to simulation is valid. Also, there is no obvious model for the evolution of discrete morphological characters.

### Comparison of support on Bayesian and Maximum Likelihood phylogenies

We used results from simulation analyses to re‐assess the empirical phylogenies: we presented topologies on which nodes with only 80% posterior probabilities or bootstrap support are presented (Figs 6, 7). For most analyses, the consensus trees bring congruence between the Bayesian and Maximum Likelihood Mk implementations, which is similar to the pattern seen with the simulated data (Brown *et al*. 2017). For one of the larger matrices analysed (Luo *et al*. 2015), resolving only nodes with over 80% support has little influence on the overall conclusions as *Haramiyavia* is still recovered outside multiturberculates in the analyses performed using the Bayesian and Maximum Likelihood implementations of the Mk model. These clades cannot be distinguished in the parsimony analyses. Similar results are seen in the analyses of the dataset from Hilton & Bateman (2006): no method supports the anthophyte hypothesis, but parsimony does not separate pteridospermous taxa from gymnosperms, seed ferns and angiosperms (O'Reilly *et al*. 2017, fig. S1). Presenting only nodes with 80% support brings congruence between all methods for the re‐analyses of Nesbitt *et al*. (2013) and Sutton *et al*. (2012). *Nyasasurus* from Nesbitt *et al*. (2013) is placed in a polytomy with basal dinosaurs in Bayesian, Maximum Likelihood and parsimony analyses (Fig. 7). Bayesian and Maximum Likelihood can only resolve three nodes, and parsimony only one node, from the reanalysis of the dataset from Sutton *et al*. (2012) in which no method can resolve the position of *Kulindroplax* (Fig. 6).

### Differences between the Maximum Likelihood and Bayesian Mk implementations

Difference in performance between Maximum Likelihood and Bayesian inference, when poorly supported nodes are collapsed, is surprising given that the primary difference between these methods is the scaling of the likelihood by the prior distribution in the Bayesian implementation. It is therefore possible that the prior distribution placed on the branch lengths may be a cause of difference in performance. As topology and branch lengths are jointly estimated in the probabilistic framework it is entirely possible that the prior distribution placed on the branch lengths is influencing the estimation of topology. A further potential cause of discrepancies between Maximum Likelihood and Bayesian inference is the partitioning of characters by the number of possible states they exhibit. MrBayes automatically partitions characters by the number of implied character states, constructing a transition matrix of the appropriate dimensions for those characters. Conversely, RAxML appears to construct a single transition matrix that is applied to all characters, which may be a contributing factor to differences between these methods. These differences may also be caused by errors introduced by the efficiency of the algorithm applied to search for the Maximum Likelihood tree or the rapid bootstrap method used to calculate support for nodes in the Maximum Likelihood topology estimate.

## Conclusions

When estimating phylogenetic relationships from morphological data, the parsimony criterion is not as accurate as the stochastic Mk model, whether clade support values are considered or not. In contrast to most previous analyses, and following Brown *et al*. (2017), we find that when accounting for clade support values the Maximum Likelihood implementation of the Mk model achieves similar overall accuracy to the Bayesian implementation of the Mk model, albeit with Maximum Likelihood producing less‐resolved phylogenies. Our simulations indicate that the Bayesian implementation of the Mk model estimates higher support for correct nodes compared to Maximum Likelihood. Therefore, we advocate the Maximum Likelihood or Bayesian implementations of the Mk model, in place of parsimony, for phylogenetic analyses based on discrete morphological data.
